# Host Plant Affects Symbiont Abundance in *Bemisia tabaci* (Hemiptera: Aleyrodidae)

**DOI:** 10.3390/insects11080501

**Published:** 2020-08-04

**Authors:** Yan-Hong Liu, M. Mostafizur Rahman Shah, Yue Song, Tong-Xian Liu

**Affiliations:** 1 College of Plant Protection, Shanxi Agricultural University, Taigu 030801, China; liuyanhong1984@126.com; 2 State Key Laboratory of Crop Stress Biology in Arid Areas, and Key Laboratory of Integrated Pest Management on the Loess Plateau of Ministry of Agriculture, Northwest A & F University, Yangling 712100, China; mostafiz.wrc@gmail.com (M.M.R.S.); songyue409@126.com (Y.S.); 3 Wheat Research Center, Bangladesh Agricultural Research Institute, Dinajpur 5200, Bangladesh

**Keywords:** whitefly, biotype B, symbiont, host plant, nutrition, essential amino acids

## Abstract

**Simple Summary:**

The nutritional contributions of symbionts facilitate herbivores’ plant utilization, promoting insects infecting and spreading on host plants. In this study we investigated the effects of host plants on the symbionts of *Bemisia tabaci* Middle East-Asia Minor 1 (MEAM1) from a nutritional aspect. We found that three host plant-adapted whitefly populations harbored the same symbiont taxa in different quantities. The amount of the primary symbiont *Portiera* decreased with increasing host-plant essential amino acid proportions in whitefly populations and even in those transferred to different host-plant species to meet the nutritional demands of whiteflies. However, the abundance of the secondary symbionts in whiteflies after host-plant-shifting for one generation showed little correlation with essential amino acid levels of host plants. It demonstrates that host-plant nitrogen nutrition—mainly, essential amino acids—influences the abundance of symbionts, especially *Portiera*, to meet whiteflies’ nutritional demands, and whiteflies manipulate their symbionts’ quantity governed by the host plant. The nutrient exchanges in symbioses involving multiple partners could provide new ideas for pest control.

**Abstract:**

Symbionts contribute nutrients that allow insects to feed on plants. The whitefly *Bemisia tabaci* Middle East-Asia Minor 1 (MEAM1) is a polyphagous pest that depends on symbionts to provide key nutrients that are deficient in the diet. Here, we established three whitefly populations on eggplants, cucumbers, and tomatoes and observed that they harbored the same symbiont taxa in different quantities. The amount of the primary symbiont, *Portiera*, decreased with increasing concentrations of host-plant essential amino acids (EAAs). Whitefly populations transferred to different plant species exhibited fluctuations in *Portiera* amounts in the first three or four generations; the amount of *Portiera* increased when whitefly populations were transferred to plant species with lower EAAs proportions. As for the secondary symbionts, the whitefly population of eggplants exhibited lower quantities of *Hamiltonella* and higher quantities of *Rickettsia* than the other two populations. The changes of both symbionts’ abundance in whitefly populations after host-plant-shifting for one generation showed little correlation with the EAAs’ proportions of host plants. These findings suggest that host-plant nitrogen nutrition, mainly in the form of EAAs, influences the abundance of symbionts, especially *Portiera*, to meet the nutritional demands of whiteflies. The results will inform efforts to control pests through manipulating symbionts in insect–symbiont associations.

## 1. Introduction

The sweet potato whitefly, *Bemisia tabaci* (Gennadius) (Hemiptera: Aleyrodidae), is a cosmopolitan pest of over 500 species of horticultural and agronomic crops in fields and greenhouses [1,2]. It feeds on phloem sap, excretes honeydew, and transmits more than 300 plant pathogenic viruses that cause over 40 different diseases in vegetables and fiber crops around the world [3,4]. *B. tabaci* is responsible for an estimated one to two billion dollars in annual losses through direct or indirect damage [5]. It is a species complex with wide-ranging genetic diversity and is composed of at least 34–36 species that were defined in the past as biotypes [6,7]. Some of the species are restricted to a small number of host plants in specific areas; others are highly polyphagous and global. Of these, *B. tabaci* Middle East-Asia Minor 1 (MEAM1) (previously termed “biotype B”) is invasive, polyphagous, viruliferous, and the most widespread and damaging group [3,8]. It adapts to a variety of host plants in different habitats.

Plant usage by polyphagous insects may be promoted by microbial symbionts that provide the hosts with nutrients or detoxify plant allelochemicals [9]. The bacterial symbionts in whiteflies are composed of a primary symbiont (P-symbiont), *Portiera aleyrodidarum* (hereafter, *Portiera*), occurring in all individuals, and one or more secondary symbionts (S-symbionts) found in some of the individuals of a population [10]. *B. tabaci* contains at least seven S-symbionts: *Hamiltonella*, *Rickettsia*, *Wolbachia*, *Cardinium*, *Arsenophonus*, *Fritschea*, and *Hemipteriphilus* [10,11,12,13].

*Portiera* is essential for the development and reproduction of whiteflies. It is located in specialized bacteriocytes in the body cavity and synthesizes nutrients, such as essential amino acids (EAAs) and carotenoids, which are missing from the phloem diet [4,14]. Although S-symbionts are facultative for whitefly survival and reproduction, they can influence whitefly fitness by mediating a resistance to parasitoids, thermotolerance, viral transmission, and hosts’ susceptibility to insecticides [15]. In addition, some S-symbionts supply vitamins, cofactors, and EAAs to their hosts [16]. The genomes of *Hamiltonella* and *Rickettsia* retain some genes involved in the biosynthesis of several EAAs, cofactors, and vitamins [4,17,18,19,20]. While the presence of these genes hints at metabolic cooperation between the S-symbionts and whiteflies [18,21,22], there have been a few studies focusing on the relationship between the amount of S-symbionts and EAAs synthesis.

Variations in the symbiont quantity influences insect fitness. In the aphid *Aphis craccivora*, the titer of its symbiont, *Buchnera*, decreases under low and high temperature stress, negatively influencing aphid reproduction [23]. Excessive symbiont density may amplify the negative or positive effects on the insect by influencing its fitness or by causing pathological damage [24]. Symbiont proliferation consumes the resources of the insect body, which are in turn obtained from the host plants. Thus, host plants may significantly influence symbiont numbers. Plants differ in their amino-acid profiles, which may affect symbiont density in polyphagous insects [25]. The fluctuation of symbiont density in whiteflies in response to differences in nitrogen nutrient levels among host plants remains unknown.

Here, we characterized the symbionts in three laboratory-grown whitefly populations that were originally derived from the same parental population and then established on eggplants, tomatoes, or cucumbers. We monitored the symbiont abundance in whiteflies transferred to natal or novel host-plant species for five generations. We also measured the amino acid compositions in the phloem sap of the three plant species and in their corresponding whitefly populations to interpret the dynamics of *Portiera* density in response to nitrogen nutrition, especially EAAs levels, in different host plants. We found that, in our laboratory-grown whiteflies, different *B. tabaci* populations harbored the same symbiont taxa in different quantities, and the amounts of *Portiera* decreased with increasing concentrations of host-plant EAAs in whitefly populations or after host-plant shifting to meet the nutritional demands of whiteflies.

## 2. Materials and Methods

### 2.1. Plants and Insects

Three economically important vegetables were used in this study: eggplants (*Solanum melongena* L. (Solanaceae) var. “Zichangqie”), tomatoes (*Solanum lycopersicum* L. (Solanaceae) var. “Florida Lanai”), and cucumbers (*Cucumis sativus* L. (Cucurbitaceae) var. “Jinchun”). Seeds were germinated, and seedlings individually transplanted into plastic pots (12 cm in diameter) with potting mix (a mixer of peat moss, vermiculite, and perlite at a 7:1:1 ratio by volume) in an insectary at 25 ± 1 °C, 60% ± 5% relative humidity (RH), and a photoperiod of 16L:8D (light:dark) at a light intensity of 1400–1725 lux [25]. Seedlings were fertilized with a dry soluble fertilizer “Harvest More 20-20-20+TE” at the rate of 1-g/L water/week. When plants had five to six leaves, some were used to rear whiteflies, and others were used to extract amino acids.

*B. tabaci* was collected from a greenhouse (Key Laboratory of Applied Entomology, Northwest A&F University, Yangling, Shaanxi, China) in 2011 and subsequently reared on tomatoes (*Solanum lycopersicum* var. “Florida Lanai”) in the insectary. The whitefly adults were identified as the MEAM1 species (GenBank accession No.: KF773139) using the mitochondrial *COI* gene [26]. Three laboratory whitefly populations were raised for more than 30 generations in cages (65 × 65 × 65 cm) on eggplants, tomatoes, and cucumbers, separately. These populations were labeled *Bemisia*-eggplant, *Bemisia*-tomato, and *Bemisia*-cucumber, respectively. They had identical genetic backgrounds. The whiteflies were maintained at 26 ± 1 °C, 65% ± 5% RH, with a photoperiod of 16L:8D and a light intensity of 1400–1725 lux [27].

### 2.2. Identification of Symbionts in Different Whitefly Populations

Total DNA for each sample was extracted from 20 female adults using the Wizard Genomic DNA Purification Kit (Promega Corporation, Madison, WI, USA). The quality of the DNA template was verified by PCR amplification of the mitochondrial *COI* gene of *B. tabaci*. Diagnostic PCR with specific primers was used to identify the symbionts infecting the whitefly populations. The *16S rRNA* gene for *Portiera*, *Hamiltonella*, *Rickettsia*, *Wolbachia*, *Cardinium*, and *Hemipteriphilus* and the *23S rRNA* gene for *Arsenophonus* and *Fritschea* were amplified. The corresponding primer sequences are listed in Table 1. PCR reactions were performed with 25-μL reaction mixtures containing 12.5-μL *Taq* Premix (TaKaRa), 1-μL forward and reverse primers (10 μM) each, 2-μL DNA template, and 8.5-μL ddH_2_O under the following cycling conditions: 95 °C for 4 min, then 35 cycles of 95 °C for 30 s, 60 °C for 30 s (except for *Wolbachia*, which was at 55 °C for 30 s), and 72 °C for 1 min, followed by a final extension at 72 °C for 10 min. Amplified DNA products were subjected to electrophoreses on 1% agarose gels, and a specific target band was regarded as diagnostic for the symbiont infection. The species of symbionts detected in the samples were further confirmed by sequencing (Invitrogen, Beijing, China).

### 2.3. Transfer of Whitefly Populations to Natal and Novel Host-Plant Species

**Figure 1:**
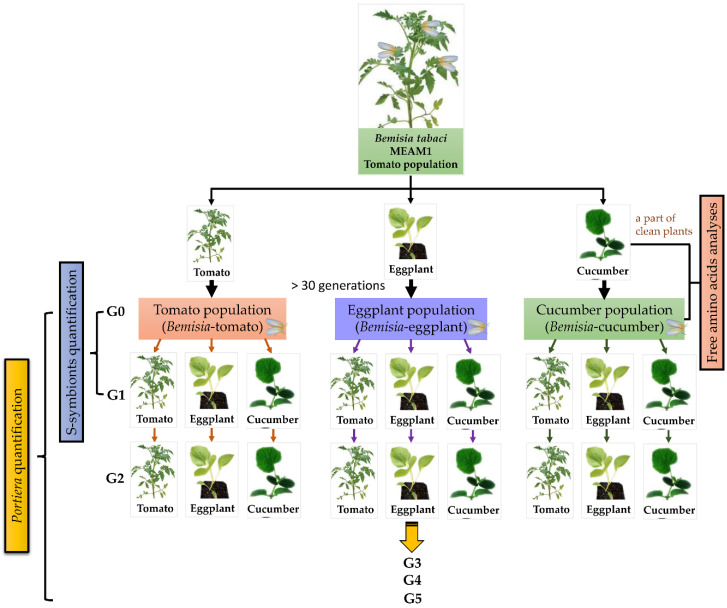
Schematic diagram of the plant switch experiments designed in this study. MEAM1: Middle East-Asia Minor 1.

From each population, about 150 five-day-old female adults (termed G0) were collected to measure the symbiont abundance. Between 150 and 200 adults were transferred to the natal or novel species for 24 h to lay eggs. The adults were then removed, and the eggs were allowed to develop. About 150 five-day-old G1 female adults were collected and the symbionts quantified. The remaining G1 adults were transferred to the three plant species to allow egg-laying for 24 h, following which, the adults were removed. About one month later, 150 five-day-old female adults (G2) were collected and stored in ethanol at −20 °C. The remaining G2 adults were transferred to fresh plants for the production of the third generation. The third (G3), fourth (G4), and fifth (G5) generation samples were collected similarly. The plant switch experiments were performed as shown in Figure 1. The G0 and G1 samples with six replicates for each treatment were used to quantify all detected symbionts, and the G2–G5 samples with six replicates for each treatment were sampled to quantify *Portiera*.

### 2.4. Quantification of Symbionts

The relative abundance of symbionts in *Bemisia*-eggplants, *Bemisia*-tomatoes, *Bemisia*-cucumbers (G0), and in the three whitefly populations transferred to the natal and novel plant species (G1–G5) was determined by quantitative PCR (qPCR). Total DNA was extracted from 20 female adults for each sample using the Wizard Genomic DNA Purification Kit, according to the manufacturer’s instructions. qPCR was performed on the iQ5 multicolor real-time PCR machine (Bio-Rad) with 20-μL mixtures containing SYBR Premix Ex *Taq* II (TaKaRa) 10 μL, forward and reverse primers (10 μM) each for 0.8 μL, DNA template 2 μL, and ddH_2_O 6.4 μL. The reaction program was 95 °C for 3 min, followed by 40 cycles of 95 °C for 10 s and 55 °C for 30 s. *Portiera*, *Hamiltonella*, and *Rickettsia* were quantified by qPCR using the number of *16S rRNA*, *16S rRNA*, and *gltA* genes, respectively. *β-actin* was used as the reference gene. The primers used here are presented in Table 1. All quantifications included six replicates, with three technical repeats for each sample. The relative amount of each symbiont was normalized using *β-actin* and calculated by the 2^-Δ*C*t^ method [36,37,38].

### 2.5. Amino-Acid Analysis

Free amino acids in plant phloem sap were collected using the EDTA-exudation technique [25,39,40]. When eggplants, tomatoes, and cucumbers grew to the 5–6-leaf stage, the second and third fully expanded fresh leaves from the top of the plant were cut. The petioles were immersed in 1-mL EDTA solution (10 mM, pH 7.0) in a 1.5-mL Eppendorf tube and incubated in darkness at 25 °C for 4 h with > 90% RH. Leaves were removed from the tubes, and any droplets attached to the petioles were tapped back into the tube. The exudates were centrifuged at 4500 *g* for 5 min at 4 °C. The supernatant was filtered through a sterile syringe filter and stored at −80 °C for further analysis. Amino acids were extracted from six biological replicates of each plant species.

Free amino acids in whiteflies were extracted using ethanol and hydrochloric acid [39]. Fifty newly emerged female adults from every population (G0) were ground in a 1.5-mL Eppendorf tube containing a 600-µL buffer of 0.1-M HCl and 100% ethanol (1:1), followed by centrifugation at 12,000 *g* for 20 min. The supernatant was filtered and kept at −80 °C for subsequent analysis. Amino acids were extracted from nine biological replicates of each whitefly population.

Amino acids in the extracts were analyzed by LC-MS. Liquid chromatography separations were carried out with a Inertsil OSD-4 C18 Column (250 mm × 3.0 mm; GL Sciences Inc., Tokyo, Japan). Amino-acid elution was performed by applying a three-step gradient: 100% A for 8 min, 0–100% B linear for 2 min, 100% B for additional 5 min, and 0–100% A linear for 1 min, holding the system at 100% A for 8 min with a flow rate of 0.3 mL/min. Mobile phase A was an aqueous solution containing 5% acetonitrile and 0.1% formic acid, and mobile phase B was 100% acetonitrile with 0.1% formic acid. The elution pattern of free amino acids was further confirmed by GC-MS using an identical column and the same procedure as described above. LTQ XL^TM^ linear ion trap mass spectrometer (Thermo Scientific, Waltham, MA, USA) was used in the positive electrospray ionization (ESI) mode. Nitrogen was used as the sheath gas (30.0 arbitrary units) and auxiliary gas (5.0 arbitrary units). The spray voltage was 4.5 kV, and the ion transfer capillary temperature was 275 °C. The amino acids were scanned and fragmented by data-dependent MS/MS. Masses of precursor and product ions and collision energy for each amino acid were based on those in Liu et al. [20]. Acquired data were processed by Xcalibur 2.1 software (Thermo Scientific, Waltham, MA, USA). Quantification was based on an external standard amino-acid mixture of a known concentration.

### 2.6. Data Analysis

All data related to symbiont abundance, and percentages of individual amino acids and entire EAAs (arginine, histidine, isoleucine, leucine, lysine, methionine, phenylalanine, threonine, tryptophan, and valine) were tested for normality using the Shapiro-Wilk test [41]. The statistical significance of differences between the experimental groups was tested with one-way ANOVA and Tukey’s honest significant difference (HSD) test at *p* < 0.05 using IBM SPSS Statistics package v. 20.0 (SPSS Inc., Chicago, IL, USA). To evaluate the effects of different factors on the amount of symbionts, two- or three-way ANOVA with Tukey’s test was used, with post facto multiple comparisons of means. Variations among amino acid–composition profiles in phloem sap and whitefly populations were measured by multiple-dimensional principal component analysis (PCA) using SAS version 9.2. Each amino-acid concentration was converted to mole percentage (mol%) and subjected to PCA. Each spot in the PCA plot represented an individual sample, and distances among the spot groups defined the variation in the amino-acid profiles among the treatments. Correlation between the abundance of *Portiera* and the proportion of EAAs in plants was tested by Spearman’s rho rank correlation coefficient analysis using SPSS 20.0.

## 3. Results

### 3.1. Different Quantities of the Same Symbiont Taxa in Three Whitefly Populations

We identified the P-symbiont *Portiera* and two S-symbionts taxa, *Hamiltonella* and *Rickettsia*, in the three whitefly populations established on eggplants, cucumbers, and tomatoes by diagnostic PCR (GenBank accession No.: KF773136–KF773138).

**Figure 2:**
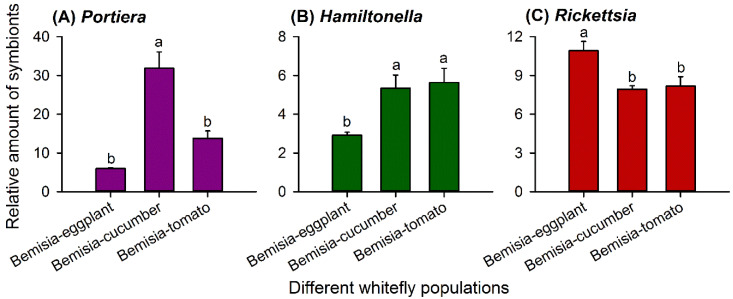
Relative abundance of *Portiera* (**A**), *Hamiltonella* (**B**), and *Rickettsia* (**C**) (mean ± SE) in whitefly populations G0 of *Bemisia*-eggplants, *Bemisia*-cucumbers, and *Bemisia*-tomatoes. The relative amount of each symbiont taxon was normalized using *β-actin* and calculated by the 2^-Δ*C*t^ method. Statistical analyses were carried out separately for each symbiont taxon; different letters above the error bars indicate significant differences for that taxon among the three whitefly populations (*p* < 0.05, Tukey’s honest significant difference (HSD) test).

The relative abundance of *Portiera* differed significantly among the three G0 populations (*F*_2,15_ = 24.532, *p* < 0.001). *Bemisia*-cucumbers harbored significantly more *Portiera* than *Bemisia*-eggplants and *Bemisia*-tomatoes (Figure 2A).

Similarly, the relative amount of *Hamiltonella* (*F*_2,15_ = 6.558, *p* = 0.009) and *Rickettsia* (*F*_2,15_ = 7.255, *p* = 0.006) differed significantly among the three populations. *Bemisia*-eggplants hosted a lower amount of *Hamiltonella* (Figure 2B) but significantly higher amounts of *Rickettsia* (Figure 2C) than the other two populations.

### 3.2. Host-Plant-Switching Affects Portiera Abundance

**Figure 3:**
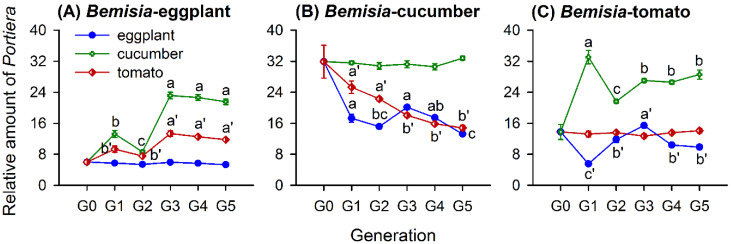
Relative amounts of *Portiera* (mean ± SE) in whitefly populations of *Bemisia*-eggplants (**A**), *Bemisia*-cucumbers (**B**), and *Bemisia*-tomatoes (**C**) transferred to natal and novel host-plant species for different generations, ranging from G0 to G5. The relative amount of each symbiont taxon was normalized using *β-actin* and calculated by the 2^-Δ*C*t^ method. Different lowercase letters and lowercase letter variants, i.e., a′, b’ and c′, around the error bars indicate significant differences among different generations after a transfer to a novel host-plant species (*p* < 0.05, Tukey’s HSD test).

The whitefly population, host-plant species, and the generation number since transfer all affected the relative amount of *Portiera*, with significant interactions among them (Table 2). In Figure 3, the relative amount of *Portiera* in the population of *Bemisia*-eggplants differed significantly across the five generations (G1–G5) when transferred to the cucumbers (*F*_4,25_ = 77.198, *p* < 0.001) and tomatoes (*F*_4,25_ = 19.407, *p* < 0.001), but on the natal plant species, it contained similar amounts of *Portiera* from G0 to G5 (*F*_5,30_ = 1.857, *p* = 0.132). The *Portiera* amounts in *Bemisia*-eggplants increased after a switch to a novel plant species (Figure 3A). It varied greatly in the first three generations and stabilized in G3, G4, and G5 on the cucumbers and tomatoes (Figure 3A).

The amount of *Portiera* in the *Bemisia*-cucumbers population showed significant variations across G1 to G5 when transferred to the eggplants (*F*_4,25_ = 15.026, *p* < 0.001) and tomatoes (*F*_4,25_ = 25.799, *p* < 0.001) and remained similar when shifted to other cucumber plants across G0 to G5 (*F*_5,30_ = 0.189, *p* = 0.964). The *Portiera* levels in *Bemisia*-cucumbers decreased over the course of five generations on the eggplants and the first three generations on the tomatoes but stabilized to similar levels among G3, G4, and G5 on the tomatoes (Figure 3B).

In *Bemisia*-tomatoes, the amount of *Portiera* varied among the five generations when transferred to the eggplants (*F*_4,25_ = 39.090, *p* < 0.001) and cucumbers (*F*_4,25_ = 16.526, *p* < 0.001), while there was no difference from G0 to G5 on the natal plant species (*F*_5,30_ = 0.255, *p* = 0.934). When shifted to the eggplants, the amount of *Portiera* decreased firstly and then increased, with large variations in the first three generations, followed by stable levels in G4 and G5 (Figure 3C). When transferred to the cucumbers, the *Portiera* amounts showed fluctuating increases in the first three generations but with similar levels in G3, G4, and G5 (Figure 3C).

### 3.3. Host-Plant-Switching Affects S-Symbiont Abundance

**Figure 4:**
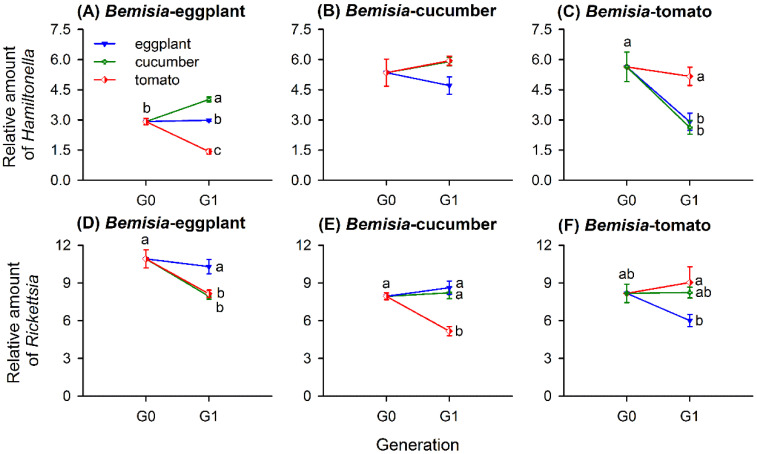
Relative amounts of *Hamiltonella* and *Rickettsia* (mean ± SE) in whitefly populations of *Bemisia*-eggplants (**A**,**D**), *Bemisia*-cucumbers (**B**,**E**), and *Bemisia*-tomatoes (**C**,**F**) transferred to natal and two novel host-plant species for one generation. The relative amounts of each symbiont taxon were normalized using *β-actin* and calculated by the 2^-Δ*C*t^ method. Different lowercase letters around the error bars of each whitefly population indicate significant difference of *Hamiltonella* or *Rickettsia* when transferred to different plant species (*p* < 0.05, Tukey’s HSD test).

When transferred to novel host-plant species, *Hamiltonella* amounts changed significantly in a natal population-dependent manner (Table 3). However, *Rickettsia* amounts changed based on both the natal whitefly population and on the novel host, with a significant interaction term (Table 3). When transferred to novel plant species, *Bemisia*-eggplants greatly changed in their *Hamiltonella* (*F*_3,20_ = 83.668, *p* < 0.001) and *Rickettsia* (*F*_3,20_ = 11.584, *p* < 0.001) quantities but did not differ when shifted to a natal plant species. Figure 4A describes *Hamiltonella* amounts in *Bemisia*-eggplants significantly increased when transferred to cucumbers but decreased greatly when moved to tomatoes. *Rickettsia* amounts reduced considerably when transferred to either cucumbers or tomatoes (Figure 4D).

Host-plant-shifting exerted no effect on *Hamiltonella* amounts of *Bemisia*-cucumbers (*F*_3,20_ = 1.887, *p* = 0.164; Figure 4B) but reduced *Rickettsia* amounts when moved to tomatoes (*F*_3,20_ = 17.368, *p* < 0.001; Figure 4E).

In *Bemisia*-tomatoes, host-plant-switching influenced the amounts of both *Hamiltonella* (*F*_3,20_ = 10.310, *p* < 0.001) and *Rickettsia* (*F*_3,20_ = 4.260, *p* = 0.018). The amount of *Hamiltonella* in *Bemisia*-tomatoes decreased when transferred to either novel plant species but exhibited similar levels on other tomatoes (Figure 4C). The amount of *Rickettsia* remained stable when transferred to the cucumbers and the natal species tomatoes but was significantly reduced when moved to the eggplants (Figure 4F).

### 3.4. Phloem Sap Amino Acid–Composition Profiles Differ among Plant Species

**Figure 5:**
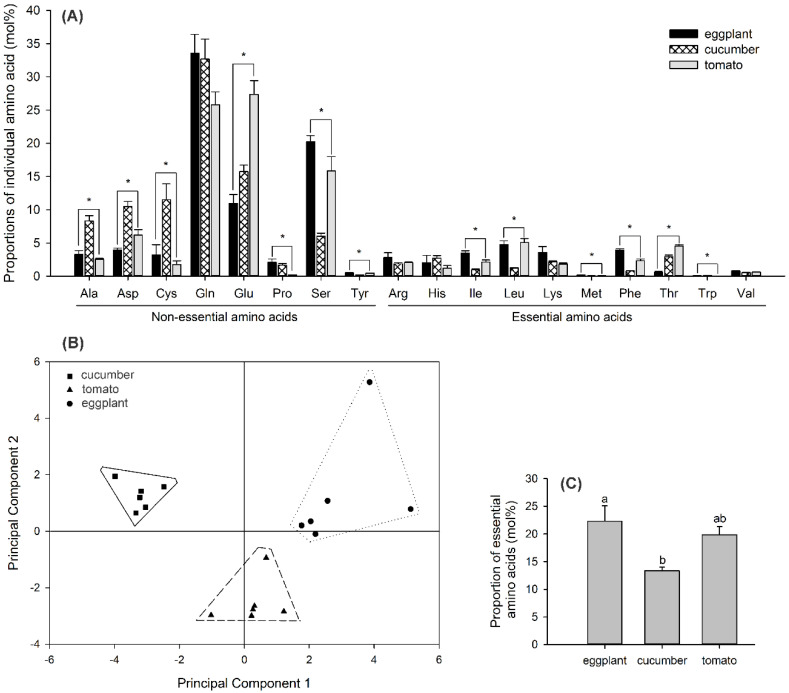
Proportions of individual amino acids (mean ± SE) (mol%; **A**), amino acid–composition analysis by principle component analysis (PCA) (**B**), and percentage of the entire essential amino acids (EAAs) in the phloem sap of eggplant, cucumber, and tomato leaves (mol%; **C**). Non-EAAs: Ala, alanine; Asp, aspartic acid; Cys, cysteine; Gln, glutamine; Glu, glutamic acid; Pro, proline; Ser, serine; and Tyr, tyrosine. EAAs: Arg, arginine; His, histidine; Ile, isoleucine; Leu, leucine; Lys, lysine; Met, methionine; Phe, phenylalanine; Thr, threonine; Trp, tryptophan; and Val, valine. Asterisk or different letters above the error bars indicate significant differences in individual amino acids or percentage of the entire EAAs among the three plant species (*p* < 0.05, Tukey’s HSD test).

There are 18 amino acids detected in the phloem sap of the eggplant, cucumber, and tomato leaves. The proportions of most individual amino acids varied significantly among the different plant species (Figure 5A), and the statistical analyses of these amino acids are shown in Table 4. For each plant species, the three most abundant amino acids were non-EAAs: glutamine (33.6%), serine (20.2%), and glutamic acid (11.0%) in eggplants; glutamine (32.7%), glutamic acid (15.7%), and cysteine (11.5%) in cucumbers; and glutamic acid (27.4%), glutamine (25.8%), and serine (15.8%) in tomatoes. Among the EAAs, isoleucine, leucine, methionine, and phenylalanine occurred in larger amounts in eggplants than in cucumbers (Figure 5A). PCA indicated lower variations in the amino acid–composition profiles within the sample replicates than among the plant species (Figure 5B). The first principal component explained 41% of the total variation and showed a strong positive association with leucine, isoleucine, phenylalanine, methionine, tyrosine, and serine levels and a negative association with alanine and aspartic acid levels. The second principal component accounted for 27% of the total variation and showed a strong positive association with tryptophan, histidine, lysine, and cysteine levels and a negative association with glutamic acid levels. The percentage of the entire EAAs differed significantly among the plant species (*F*_2,15_ = 6.065, *p* = 0.012). Eggplants had the highest percentage of the entire EAAs, cucumbers had the lowest, and tomatoes had an intermediate percentage that was not significantly different from either (Figure 5C). The percentage of the entire EAAs in the three plant species correlated negatively with the *Portiera* amount in the corresponding whitefly populations (Spearman’s rho: *r* = −0.473, *p* = 0.048).

### 3.5. No Significant Differences in Amino Acid–Composition Profiles among Whitefly Populations

**Figure 6:**
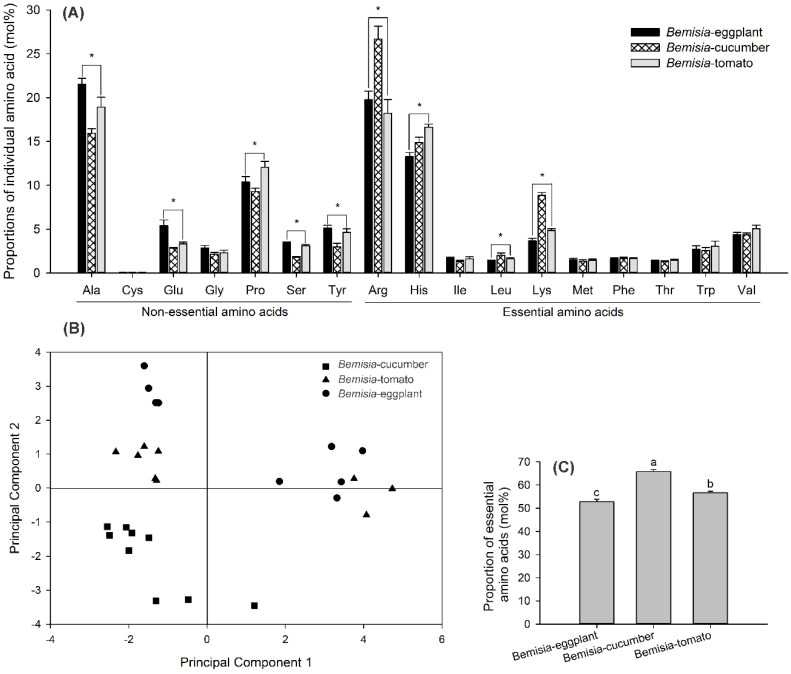
Proportions of individual amino acids (mean ± SE; mol%; **A**), amino acid composition–profile analysis by PCA (**B**), and percentage of the entire EAAs in three whitefly populations (G0), termed *Bemisia*-eggplants, *Bemisia*-cucumbers, and *Bemisia*-tomatoes (mol%) (**C**). Asterisks or different letters above the error bars indicate significant differences in individual amino-acid levels or percentage of the entire EAAs among the whitefly populations (*p* < 0.05, Tukey’s HSD test).

A total of 17 amino acids detected in the whitefly populations. The proportions of most individual amino acids differed significantly among the whitefly populations (Figure 6A), and the statistical analyses of these amino acids are shown in Table 4. PCA revealed no significant differences in the amino acid–composition profiles among the three whitefly populations. *Bemisia*-cucumbers were separable from *Bemisia*-eggplants and *Bemisia*-tomatoes, but the latter two populations could not be distinguished based on the amino-acid profiles (Figure 6B). The first principal component accounted for 36% of the total variation and demonstrated a strong positive association with isoleucine, tryptophan, proline, and tyrosine levels and a negative association with arginine levels. The second principal component explained 20% of the total variation and had a strong positive association with alanine, glutamic acid, and serine levels and a negative association with leucine and lysine levels. However, the percentage of the entire EAAs differed significantly among the whitefly populations (*F*_2,24_ = 88.777, *p* < 0.001). *Bemisia*-cucumbers contained a significantly higher percentage of the entire EAAs than *Bemisia*-tomatoes and *Bemisia*-eggplants. *Bemisia*-eggplants had the lowest percentage of the three populations (Figure 6C).

## 4. Discussion

Host-plant nutrition is important for the performance of herbivorous insects, especially sap suckers [25]. These insects are highly dependent on nutrient provisioning by intracellular symbionts [42]. Our study indicated that host plants substantially influence the abundance, but not the taxa, of symbionts in *B. tabaci* MEAM1. The S-symbionts infecting our study population are *Hamiltonella* and *Rickettsia*, which are comparable to those documented in the same *B. tabaci* species in China, Israel, and Brazil [6,11,12,43]. We established three experimental populations from the same parental population and detected the same symbiont taxa in all three experimental populations. Usually, the change of the symbiont taxa in the insect host is associated with horizontal transmission and environmental factors. A new infection of S-symbionts is through occasional events of horizontal transmissions among different insect species or different individuals mediated by plant, parasitoids, or copulation [44,45,46,47,48]. High temperatures can eliminate the mutualistic partners from insect hosts; many bacteriocyte-associated symbionts have reduced densities or are lost entirely when the insect host is exposed to high temperatures [49,50,51]. Our experimental populations did not contact other infectious populations, species, or host plants fed by other infectious populations and were kept in controlled conditions in the laboratory. Accordingly, we speculate that coinfections of new symbionts or the elimination of existing symbionts might not occur without contact with different infectious populations or without changing the environmental factors.

The host-plant species strongly affected the abundance of different symbionts in *B. tabaci*. In our study, the amount of symbionts, including *Portiera*, *Hamiltonella*, and *Rickettsia* differed significantly in the three whitefly populations grown on different host-plant species (Figure 2) and after transferring to novel host-plant species (Figure 3 and Figure 4). Likewise, previous studies reported that the relative amount of *Portiera*, *Hamiltonella*, *Rickettsia*, and *Cardinium* in MEAM1 and *Portiera* and *Hamiltonella* amounts in *B. tabaci* Mediterranean (MED) (previously termed “biotype Q”) were greatly influenced by the host plants, even with the same parental whitefly [36,52]. The host-adapted MEAM1 population on the cucumbers harbored more *Portiera* than the populations on the cabbages and cotton, and the cabbage population harbored more *Hamiltonella*, *Rickettsia*, and *Cardinium* than the cucumber and cotton populations [36]. However, the MED species of the cabbage population harbored more *Portiera* than those of the cucumber and cotton populations, and the cucumber and cabbage populations harbored more *Hamiltonella* than that of the cotton population [52]. In the green peach aphid *Myzus persicae*, the quantities of the symbionts *Buchnera* and *Serratia symbiotica* differed significantly among the aphid populations that fed on different host-plant species [25]. The amount of *Buchnera* differed significantly in populations of the cotton aphid *Aphis gossypii*, that had been reared on different host plants over a long duration [53]. Thus, long-term associations between phloem sap-sucking insects and their host plants affect the amount of symbionts that they host.

Although the total nitrogen content of the plant tissue is commonly used as the index of the nutritional value of plants for insects, the nitrogen quality—mainly, the ten EAAs—can be of crucial nutritional importance [54]. We observed that amino acids in different host-plant phloem saps predominantly comprised non-EAAs (EAAs: 13.4–22.3%, Figure 5A,C), but amino acids in different whitefly populations mainly comprised EAAs (51.3–65.0%, Figure 6A,C). PCAs showed that amino acid–composition profiles differed among host-plant species (Figure 5B) but not among the whitefly populations (Figure 6B). These data imply an essential role for the symbionts in providing hosts with EAAs. Eggplant was a more nutritious host plant—due to the higher proportion of EAAs—than tomato and cucumber, and that cucumber was the least nutritious host plant, with the lowest percentage of EAAs (Figure 5C). In an earlier study, we showed that *B. tabaci* MEAM1 had better fitness, i.e., a shorter developmental time and higher immature survival rate, on eggplants than on other plants [27]. Studies consistently indicate that eggplant is the most suitable host plant for different populations of *B. argentifolii* (*B. tabaci* MEAM1) among cucumber, sweet pepper, tomato, and garden bean, based on life-table analyses [55,56]. While the suitability for tomatoes and cucumbers differed depending on *B. argentifolii* populations and tested cultivars [55,56]. For the Japanese population, tomatoes appeared to be the least suitable host plant among eggplants, cucumbers, and sweet peppers [55], while the Florida *B. tabaci* population that fed on tomatoes had a higher intrinsic rate of increase than that on cucumbers [56]. These results highlight the importance of nitrogen as a limiting factor in the nutrition of whiteflies.

The nitrogen nutrition of the host plant impacts the population density of symbionts. We observed that the whitefly population established on eggplants with a higher EAA proportion harbored a lower abundance of *Portiera* (Figure 2 and Figure 5). After host-plant-shifting, the *Portiera* amount in whiteflies increased when transferred to new plant species with lower percentages of EAAs (Figure 3 and Figure 5). It demonstrates that the whitefly could manipulate its symbiont density to compensate for the deficiencies in the nitrogen nutrition of the host plant. The proliferation of symbionts relies on the consumption of resources from the insect body; consequently, infection densities may be significantly influenced by the nutritional condition of host plants fed by the insect [54]. Previous studies also indicate the important role of host-plant or diet nutrition in symbiont regulation [9,24,54,57]. Studies on aphids show that *Buchnera* densities in *Acyrthosiphon pisum* and *M. persicae* increase with increasing nitrogen levels in the aphid diet or host plants [19,20], while *Aphis fabae* has elevated *Buchnera* densities on *Lamium purpureum*, with a lower amino acid content than *Vicia faba* [57]. These inconsistencies among whitefly and aphids and among different aphid species demonstrate that the density of the P-symbionts is not maintained at a fixed association with host plants and can vary, possibly to meet the nutritional demands of the insect host, and, ultimately, influences insects’ fitness [24,58]. Even so, we should find out some more evidence to support fine-tuned symbiont densities in *B. tabaci* MEAM1 in response to the changes of nitrogen nutrition in plants or diets in our future work. Symbionts control the host-plant utilization of insects by regulating the host plant-derived carbon and nitrogen inputs to bacteriocytes [42,53]. Compared with the bacteriocytes of aphids, which are inhabited only by *Buchnera*, in whiteflies, most of the S-symbionts share bacteriocytes with *Portiera* [43]. Therefore, *Portiera* competes with the coresident S-symbionts for nutrients and space, which may explain the different responses observed with *Buchnera* in aphids. The impacts of symbionts on their insect hosts are determined by the balance between the cost of nutrients consumed by the symbionts and the benefits of nutrients released back to the host [20]. Therefore, elucidating the mechanisms underlying the nutrient exchange in symbioses involving multiple symbionts could provide new ideas for pest control.

Since host-plant-shifting, the relative amount of *Portiera* in whitefly populations changed on the new host-plant species but did not differ on the natal plant species with which the whitefly populations had over 3 years of interactions (Figure 3). Judging from the dynamics of the *Portiera* amounts, we assumed that, after transferring to a new host-plant species for many more generations, *Portiera* in whiteflies will reach an amount similar to the corresponding whitefly populations established on this plant species. When *A. gossypii* are transferred to novel host plants, *Buchnera* densities fluctuated in the first two generations and became stable if they continued feeding on that plant species [53]. Therefore, variations in the *Portiera* abundance after transferring to new host plants may reflect a process of nutritional compensation critical to whitefly survival, since it confers whiteflies with the ability to expand their host-plant range.

With regard to the S-symbionts, both *Hamiltonella* and *Rickettsia* genomes keep most of genes involved in synthesis of two EAAs: phenylalanine (Phe) and lysine (Lys), which are missing in the genome of *Portiera* [4,17,18,19,20,21]. In the present study, lower quantities of *Hamiltonella* and higher quantities of *Rickettsia* were found in the whitefly populations on eggplants, a more-nitrogen diet (Figure 2 and Figure 5); the former showed similar changes with *Portiera* amounts in response to different host plants. In *A. pisum*, the population density of the S-symbiont *S. symbiotica* increased when reared on low-nitrogen diets, indicating distinct regulatory mechanisms with the P-symbiont *Buchnera* [24]. However, similar with *Buchnera*, the density of *Regiella insecticola* and *Hamiltonella defensa* in *A. fabae* increased in *L. purpureum*, a low-nitrogen diet [57]. Therefore, variations of the S-symbionts in response to diet nutrition may be attributed to differences between symbionts in metabolic capabilities or access to insect nutrients. In our study, *Rickettsia* showed an opposite trend in abundance with *Hamiltonella*, which was inconsistent with the previous study [36]. The relative amounts of different S-symbionts, i.e., *Hamiltonella*, *Rickettsia*, and *Cardinium*, had the same trend in different whitefly populations established on cotton, cucumbers, and cabbages [36]. The discrepancy may be caused by the whitefly genetic background, interactions between S-symbiont taxa, and population inertia. Recent research indicated that *Hamiltonella* can synthesize biotin; biotin provisioned by whitefly horizontally transferred genes (HTGs) affects the survival and fecundity of adult whiteflies [22]. A *Hamiltonella* deficiency reduced the level of B vitamin but not EAAs and affected the sex ratio, so this symbiont affects sex ratios in *B. tabaci* MEAM1 by regulating the fertilization and supplying B vitamins [21]. Except for the nutritional functions, S-symbionts may confer ecologically important traits, e.g., a resistance to parasitoids, thermotolerance, viral transmission, and hosts’ susceptibility to insecticides [15]. *B. tabaci* MED infected with *Hamiltonella* grow faster under nutritional stress conditions [14]. MEAM1 species infected with *Rickettsia* were substantially fitter than uninfected ones: they produced more offspring, especially daughters, had a higher survival to adult, and developed faster [59,60]. We found that after host-plant-shifting for one generation, the changes of both S-symbionts’ abundance showed little correlation with the EAA proportions of the host plants (Figure 4 and Figure 5). Therefore, many generations of host-plant-shifting need to be observed to clarify the correlation. We speculate that the S-symbionts infecting our whitefly populations may confer nutrition or fitness advantages by complicated interactions among *Hamiltonella*, *Rickettsia*, and *Portiera* and even insect hosts when feeding on different host plants.

Host plants influence symbionts in two major ways: nutrients and phytotoxins [53]. Phytotoxins, or secondary metabolites, can exert positive or negative effects on the proliferation of symbionts. Many plant secondary metabolites have bacteriostatic and bactericidal activities [61]. These vary across host plants [53,61]. Future studies may explore the effects of phytotoxins on the abundance of symbionts to explain the changes of symbionts’ abundance after host-plant-shifting.

## 5. Conclusions

Host plants affect the abundance, but not the taxa, of symbionts in three host-plant-adapted whitefly populations. The abundance of the P-symbiont *Portiera* in the whitefly populations decreased with the increase of the nitrogen levels of the host plant. Whitefly populations transferred to different host-plant species exhibited fluctuations in *Portiera* amounts in the first three or four generations; the amount of *Portiera* increased when the whitefly populations were transferred to lower-nitrogen nutrition plant species. The amount of S-symbionts changed based on a natal population or both the natal population and novel host species after host-plant-shifting, exhibiting little correlation with the nutritional quality of the host plants for just one generation. The whitefly could manipulate its symbionts’ quantity governed by the host plants. Further investigations are required to better understand the cooperative metabolism in multi-partner associations in insect, symbionts and host plants.

## Figures and Tables

**Table 1:** Primers used for symbiont identification and quantification.

Organism and Target Gene	Primer Sequences (5′ to 3′)	Product Length (bp)	References
**Diagnostic PCR**			
*Bemisia tabaci* *mtCOI*	C1-J-2195: TTGATTTTTTGGTCATCCAGAAGT L2-N-3014: TCCAATGCACTAATCTGCCATATTA	800	[26]
*Portiera* *16S rRNA*	Por-F: TGCAAGTCGAGCGGCATCAT Por-R: AAAGTTCCCGCCTTATGCGT	1000	[28]
*Hamiltonella* *16S rRNA*	Ham-F: TGAGTAAAGTCTGGGAATCTGG Ham-R: CCCGGGAACGTATTCACCGTAG	1000	[28]
*Rickettsia* *16S rRNA*	Rb-F: GCTCAGAACGAACGCTATC Rb-R: GAAGGAAAGCATCTCTGC	900	[29]
*Wolbachia* *16S rRNA*	Wol-16S-F: CGGGGGAAAAATTTATTGCT Wol-16S-R: AGCTGTAATACAGAAAGTAAA	700	[11,30]
*Cardinium* *16S rRNA*	Ch-F: TACTGTAAGAATAAGCACCGGC Ch-R: GTGGATCACTTAACGCTTTCG	400	[31]
*Hemipteriphilus* *16S rRNA*	Hem-F: GCTCAGAACGAACGCTRKC Hem-R: TTCGCCACTGGTGTTCCTC	670	[15]
*Arsenophonus* *23S rRNA*	Ars23S-1: CGTTTGATGAATTCATAGTCAAA Ars23S-2: GGTCCTCCAGTTAGTGTTACCCAAC	900	[32]
*Fritschea* *23S rRNA*	U23F: GATGCCTTGGCATTGATAGGCGATGAAGGA 23SIGR: TGGCTCATCATGCAAAAGGCA	600	[11,33]
**qPCR**			
*B. tabaci* *β-actin*	qActinF: TCTTCCAGCCATCCTTCTTG qActinR: CGGTGATTT CCTTCTGCATT	130	[34,35]
*Portiera* *16S rRNA*	qPor-F: TAGTCCACGCTGTAAACG qPor-R: AGGCACCCTTCCATCT	229	[36]
*Hamiltonella* *16S rRNA*	qHam-F: GCATCGAGTGAGCACAGTTT qHam-R: TATCCTCTCAGACCCGCTAGA	243	[35,36]
*Rickettsia* *gltA*	qgltA-F: AAAGGTTGCTCATCATGCGTT qgltA-R: GCCATAGGATGCGAAGAGCT	80	[34,35]

**Table 2:** Effects of the *B. tabaci* population, host plant, and generation number since host-plant-shifting on *Portiera* abundance, determined by three-way ANOVA. df: degrees of freedom.

Effect	*Portiera* Amount			
	df	Mean Square	*F*	*p*
Population (A)	2	2713.206	943.388	<0.001
Host plant (B)	2	5202.316	1808.857	<0.001
Generation (C)	4	77.291	26.874	<0.001
A × B	4	103.224	35.891	<0.001
A × C	8	78.090	27.152	<0.001
B × C	8	75.856	26.375	<0.001
A × B × C	16	62.033	21.569	<0.001

**Table 3:** Effects of the *B. tabaci* population and host plant since host-plant-shifting on *Hamiltonella* and *Rickettsia* abundance, determined by two-way ANOVA.

Effect	*Hamiltonella* Amount				*Rickettsia* Amount			
	df	Mean Square	*F*	*p*	df	Mean Square	*F*	*p*
Population (A)	2	29.861	28.377	<0.001	2	16.368	11.706	<0.001
Host plant (B)	2	3.084	2.931	0.064	2	8.526	6.098	0.005
A × B	4	12.951	12.307	<0.001	4	18.938	13.544	<0.001

**Table 4:** Statistical analyses of individual amino acids detected from plant phloem sap and from whitefly populations.

Amino Acid	Plant Leaves		Whitefly	
	*F*_2,15_ Value	*p*-Value	*F*_2,24_ Value	*p*-Value
Ala	30.19	<0.001	12.08	<0.001
Asp	23.10	<0.001	undetected	
Cys	9.93	0.002	0.45	0.642
Gln	2.61	0.107	undetected	
Glu	30.52	<0.001	12.85	<0.001
Gly	undetected		1.61	0.216
Pro	9.20	0.003	6.26	0.005
Ser	27.04	<0.001	32.64	<0.001
Tyr	16.12	<0.001	8.32	0.001
Arg	1.40	0.277	10.88	<0.001
His	1.11	0.354	12.21	<0.001
Ile	20.62	<0.001	1.25	0.299
Leu	19.76	<0.001	3.73	0.035
Lys	3.40	0.061	85.69	<0.001
Met	35.09	<0.001	0.44	0.646
Phe	68.71	<0.001	0.02	0.984
Thr	80.39	<0.001	0.54	0.588
Trp	13.22	<0.001	0.12	0.890
Val	2.65	0.104	1.19	0.316
